# Economics of hypothalamic obesity in patients with craniopharyngioma and other rare sellar/suprasellar tumors

**DOI:** 10.1007/s10198-025-01786-3

**Published:** 2025-05-09

**Authors:** Julian Witte, Nicolas Touchot, Bastian Surmann, Kylie Braegelmann, Mathias Flume, Julia Beckhaus, Carsten Friedrich, Hermann L. Müller

**Affiliations:** 1 Vandage GmbH, Detmolder Straße 30, 33604 Bielefeld, Germany; 2 Rhythm Pharmaceuticals, 222 Berkeley Street, Boston, MA 02116 USA; 3 Gene Access GmbH, Seeweg 26, 44263 Dortmund, Germany; 4 Department of Pediatrics and Pediatric Hematology/Oncology, University Children’s Hospital, Carl von Ossietzky Universität, Klinikum Oldenburg AöR, Rahel-Straus-Straße 10, 26133 Oldenburg, Germany

**Keywords:** Hypothalamic obesity, Tumor/treatment related aHO, Rare sellar/suprasellar tumors, Craniopharyngioma, Endocrinology

## Abstract

**Background:**

Rapid and abnormal weight gain resulting in severe persistent obesity due to physical, tumor- and/or treatment-related damage to the hypothalamus, is called acquired hypothalamic obesity (aHO), and is often linked to craniopharyngioma and/or sellar/suprasellar tumors. Here, we examine the healthcare resource use (HCRU) and costs of aHO following treatment of these tumors.

**Methods:**

We used a retrospective matched cohort design with German statutory health insurance data on 5.42 million people from 2010 to 2021. We applied a novel three-step approach using diagnostic and prescription data to identify patients with treatment- or tumor-related (TTR)-aHO. We measured HCRU and costs across hospitalizations, outpatient visits, visits per specialist group, and outpatient prescription medications.

**Results:**

Compared to non-HO obesity, TTR-aHO is associated with increased hospitalizations, increased outpatient physician visits, and increased prescription use in the two years after incident tumor surgery/radiotherapy. Excess costs of TTR-aHO are €19,900 per patient in the first year and €10,700 in the second, driven by inpatient costs. Cost-intensive hormone replacement therapies like somatropin lead to a sharp increase in prescription costs in the second year.

**Conclusions:**

This study provides the first real-world analysis of TTR-aHO economics, emphasizing the importance of HCRU and costs for decision-making. Previously, economic evaluations have been missing due to the lack of a standard method to identify patients with aHO in retrospective studies. Using a new identification approach, the study reveals that TTR-aHO poses a significant burden in extensive treatment requirements for patients and high related costs for the healthcare system.

**Supplementary Information:**

The online version contains supplementary material available at 10.1007/s10198-025-01786-3.

## Background

Obesity is associated with higher risk for several serious health conditions, such as hypertension, type 2 diabetes, hypercholesterolemia, coronary heart disease (CHD), stroke, asthma, and arthritis [1, 2]. Several studies offer retrospective or prospective estimates of the degree of disease incidence that can be linked to obesity, and of the magnitude of associated direct medical costs [3, 4]. However, these studies often overlook obesity types that are uncommon and not tied to lifestyle [5, 6].

Acquired tumor/treatment-related hypothalamic obesity (TTR-aHO), often resulting from hyperphagia and from low energy expenditure, is abnormal weight gain commonly described in the context of craniopharyngioma and its treatment, but can also occur due to sellar/suprasellar tumors of different histology, irradiation, trauma, inflammation or surgical insult to the hypothalamus [5, 6]. Survivors of hypothalamic and sellar/suparasellar tumors are at risk for clinically relevant hypothalamic syndrome impacting health and health-related quality of life by increased morbidity [9, 10]. The most common causes of acquired hypothalamic damage are space-occupying lesions, such as craniopharyngiomas and pituitary macroadenomas, with sellar/suprasellar extension and invasion of the hypothalamic nuclei [8]. Hypothalamic damage can be caused by the tumor or subsequent surgical and/or radiooncological treatment [11, 12].

TTR-aHO is a rare disease with limited available data on its epidemiology. Most published estimates are based on the most common associated diagnosis, craniopharyngioma, which accounts for over 50% of all TTR-aHO cases [6, 13]. Recently published research using a cohort identical to this one estimated an annual incidence of TTR-aHO in Germany of between 0.7 and 1.7 cases per 1,000,000 persons [14]. Clinical management poses challenges despite available treatment options like medication and lifestyle modifications [15, 16]. Understanding the patient’s individual burden and articulating the systemic impact of the disease are critical steps in developing treatment guidelines and assessing new therapies.

Although cost estimates for non-hypothalamic-related obesity (non-HO) are available in Germany [17], they are not applicable to patients with TTR-aHO. Until now, analysis of healthcare service use and the associated costs specific to TTR-aHO was impeded by the lack of reliable data on the condition. Developing a clear and reproducible algorithm to identify patients with TTR-aHO and related tumors allows us to assess the healthcare resource usage and costs and to compare them with those of patients with non-HO-related obesity. In doing so, we provide insight into the economic burden of this rare endocrine disease on the healthcare system.

## Methods

### Study design and database

Our study uses a retrospective matched cohort design and has been conducted following applicable subject privacy requirements and the guiding principles of the Declaration of Helsinki of 1964. Analyses were based on anonymized routinely collected claims data from German Statutory Health Insurance (SHI), a system which insures 88% of the German population [18]. GWQ ServicePlus AG, a joint venture of medium-sized SHI funds in Germany, provided the data. The dataset comprises information on 5.42 million people insured at 19 SHI funds, representing 6.3% of the total German SHI population. Based on a comparison with official statistics published by the German Health Ministry on SHI (“KM6”), the data set is representative of the German SHI population in terms of age and gender distribution [19]. Analyses were based on data covering the period 2010–2021. We used the years 2011–2019 for patient identification, allowing for a 1-year wash-in and 2-year post-observational period. Diagnostic data include all diagnoses documented during physician outpatient contacts and patient hospital stays. Laboratory or clinical parameters were not included. A general description of the claims database in the German setting is available from Swart et al. [20].

### Study population

The initial data set represents all persons in the database aged between 0 and 100 years in the years 2010–2021. We only considered patients with at least 36 consecutive months of observational period (1 year before index hospitalization for incidence validation, 2 year after index hospitalization for aHO validation) in the data set. Individual information on birth year was aggregated to 5-year intervals in the anonymization process. We identified patients with TTR-aHO using a three-step approach. First, we selected all patients hospitalized with an incident primary- or secondary-discharge tumor diagnosis known to potentially lead to TTR-aHO (“index hospitalization”) and incident inpatient brain surgery/radiotherapy. Incidence was validated based on the preceding 12 months without respective tumor diagnosis and brain surgery/radiotherapy. Second, we applied incident obesity diagnosis (International Classification of Diseases, 10th Revision, German Modification [ICD-10-GM] E66.x, E67.x, E68, R63.2) within 12 months of index hospitalization as a mandatory criterion for TTR-aHO-patient definition. Third, we applied arginine vasopressin deficiency (AVP-D; ICD-10-GM E23.0, E23.2, E23.3, E23.6, E23.7, P80.x, P81.x) and a desmopressin prescription (Anatomical Therapeutic Chemical-Classification [ATC]-code H01BA02) within the post-observational period as TTR-aHO-validation criteria. The criteria for defining TTR-aHO and validation may involve different medical consultations or hospital admissions.

We chose AVP-D as a validation criterion for TTR-aHO due to a higher likelihood of post-surgical weight gain and more significant damage to the hypothalamus, especially in the posterior region, as seen on magnetic resonance imaging (MRI) scans. While AVP-D does not directly cause TTR-aHO, it can act as a marker of endocrine damage to the hypothalamus. Diagnosing AVP-D can be complex. Since all patients with AVP-D require treatment, the use of desmopressin is set as a further validation criterion to indicate hormone replacement therapy.

Definition of TTR-aHO and other relevant patient identification criteria were made based on literature following review of medical records of patients with TTR-aHO treated in a large German pediatric clinic with > 100 cases of TTR-aHO per year. However, as this study relies on anonymized claims data from a specific data source, we were not able to manually review individual cases nor were we able to test our algorithm on another database.

To distinguish between acquired and genetic risks for HO, individuals with a history of Prader-Willi Syndrome (ICD-10-GM Q87.1) were excluded from the study. A more detailed description of the epidemiological estimates following this TTR-aHO patient identification strategy can be found elsewhere [21].

### Healthcare resource utilization and cost estimates

We evaluated healthcare resource utilization (HCRU) and associated costs at an individual patient level and then aggregated across all TTR-aHO observations. Resources included hospitalizations, total outpatient physician visits (including lab visits and visits within structured care programs), visits per specialist group, and outpatient prescription medications. Notably, we were not able to assess inpatient medication dispensing separately, because these are bundled in a lump sum in German claims data. Exceptions are made for expensive medications, where an additional fee is paid if they are dispensed in a hospital setting. However, for this analysis, such high-priced drugs were excluded.

We tracked HCRU for each interaction or prescription, and unadjusted costs were also provided at this granularity in the dataset. These utilization and cost metrics are available on a daily basis. For this analysis, we aggregated them at the quarterly and annual levels following index hospitalization. Specifically focusing on medication courses, we examined the utilization and costs associated with hormone replacement therapies, as these are among the most common therapies for patients with TTR-aHO. The drugs under consideration were desmopressin (ATC H01BA02), hydrocortisone (ATC H02AB09), testosterone (ATC G03BA03), somatropin (ATC H01AC01), levothyroxine-sodium (ATC H03AA01) as well as the female sex steroids including estrogens (ATC G03C), progestogens (ATC G03D), and their combinations (ATC G03F).

### Matching, outcomes and statistical analyses

Based on identified patients with incident TTR-aHO, we descriptively evaluated HCRU and costs within two years after index hospitalization. We reported means and standard deviations (SD). Based on exact 1:10 matching, we compared HCRU and costs of patients with TTR-aHO to matched patients with non-HO-obesity. Matching criteria comprised five-year age groups, sex, and—as a proxy for morbidity—Rx prescription costs in the year preceding the aHO-related index hospitalization. We compared HCRU and cost parameters with those of the TTR-aHO cohort to the exact date. Patients with non-HO-related obesity were identified based on one observed prevalent obesity diagnosis (ICD-10 E66) within the observational period. Patients with tumor diagnosis (ICD-10 C or D) could not be included as an obesity patient. Comparisons from the general population met the inclusion criteria for matching when their history did not include cancer or obesity.

We evaluated matching quality using the standardized mean difference (SMD) across all matching criteria. A SMD > 10% was considered to indicate relevant covariate imbalance. Excess HCRU and costs were calculated for eight quarters following index hospitalization. We followed STROBE criteria in reporting study results. We performed analyses with R (Version 4.1.3), a standard and open-access statistical-computing programming language. We used the MatchIt package for matching.

## Results

### Study population

**Fig. 1:**
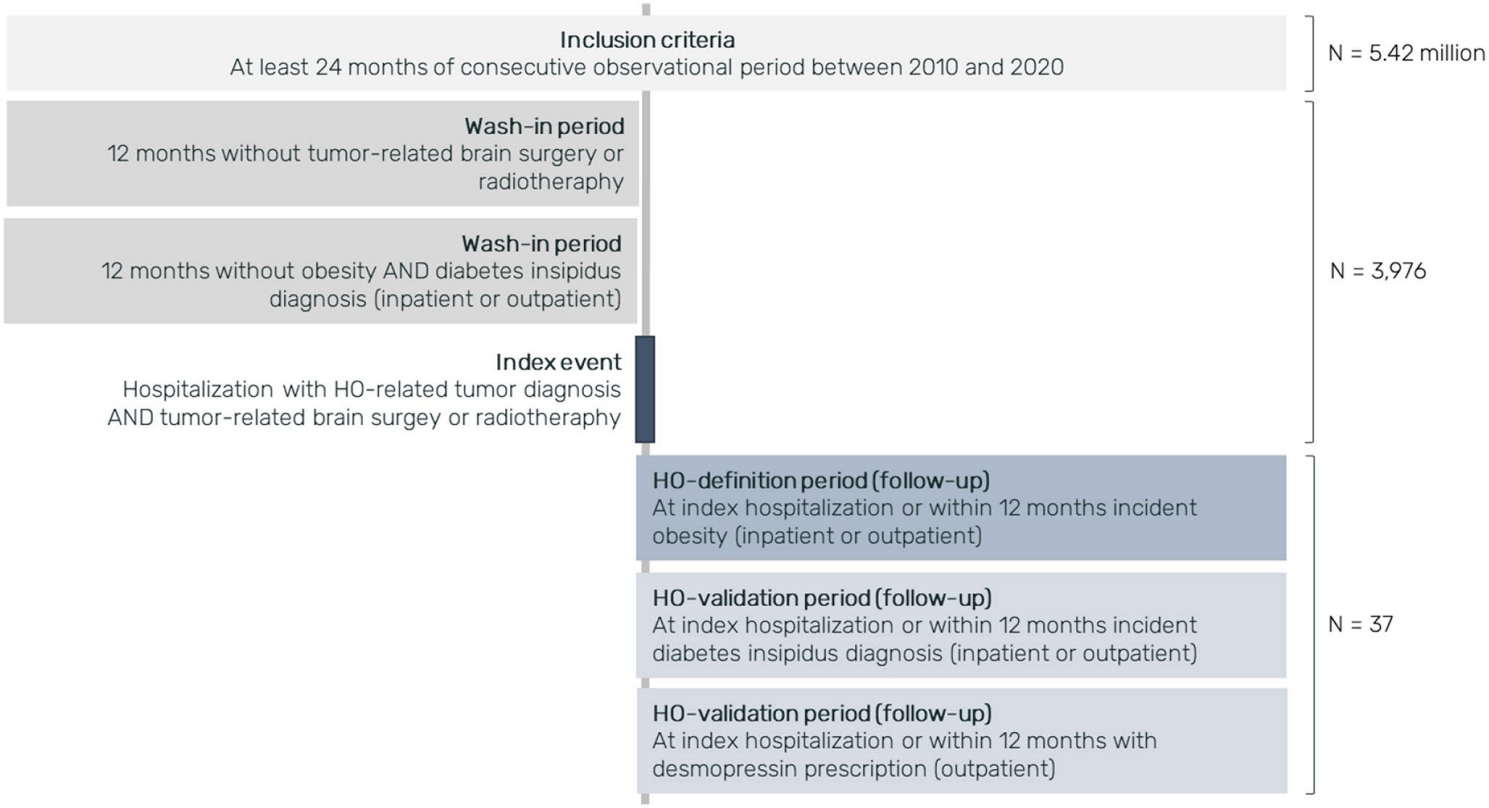
Flow chart of patient inclusion and follow up

Based on the database of 5.42 million persons from 2011 to 2019, between 3.93 and 4.41 million persons met the inclusion criteria annually. Over the time period, we identified a total of 3,976 patients with index hospitalization for treatment of tumors potentially leading to TTR-aHO. Of these, a total of 37 patients (3–7 patients annually, see Table 1) experienced an index hospitalization for tumor treatment and could be validated with TTR-aHO (Fig. 1). Patients were more often female (59.5%), with a mean age of 38 years at index hospitalization (standard deviation [SD]: 18.0 years).

**Table 1:** Study population, validated aHO population, estimated aHO incidence rate and aHO patient characteristics

	2011	2012	2013	2014	2015	2016	2017	2018	2019	2011-19
Number of patients (N) in database, Million	3.93	4.02	4.06	4.11	4.18	4.27	4.34	4.37	4.41	5.42
Number of patients with index hospitalization (n) in database	422	431	448	415	415	479	436	464	466	3,976
Incident aHO cases (n)	5	5	3	7	3	3	3	3	5	37
aHO incidence rate (/1,000,000)	1.3	1.2	0.7	1.7	0.7	0.7	0.7	0.7	1.1	6.8
Mean age (SD)	40.0 (11.4)	44.0 (18.5)	44.3 (10.5)	38.4 (25.5)	26.3 (15.3)	47.3 (20.2)	31.7 (25.7)	22.7 (20.2)	40.0 (10.4)	38.0 (18.0)
Age groups (%), < 20 years	0	20	0	14	33	0	33	67	0	16.2
Age groups (%), 20–64 years	100	40	100	57	67	100	33	33	100	70.3
Age groups (%), 65 + years	0	40	0	29	0	0	33	0	0	13.5
Share females (%)	40	60	33	86	100	33	33	67	60	59.5

### Healthcare resource utilization

Following the index event, patients with TTR-aHO in the study had an average of 5.1 hospitalizations in the first year, decreasing to 3.3 average hospitalizations in the second year (Table 2). Most common primary diagnoses in follow-up hospitalizations were tumor-specific diagnoses. Also common were diagnoses of other pituitary disorders (ICD-10-GM E23.6) and AVP-D (ICD-10-GM E23.2). A total of 18% of all patients with TTR-aHO had an emergency room readmission associated with the underlying tumor diagnosis within 90 days of the index event (see supplementary material). In terms of excess resource use, in the first year, patients with TTR-aHO had an average of 4.6 hospitalizations more than patients with non-HO-obesity (+ 2.8 hospitalizations in the second year).

Average total outpatient visits declined over time, with an average of 28.9 visits in the first year and 24.1 visits in the second year. Mean number of general practitioner contacts were 6.6 in the first year after index hospitalization and 6.7 in the second year. The mean utilization of endocrinologists/diabetologists was 1.4 contacts (year 1 post index hospitalization) and 1.1 (year 2), respectively. Within the first year, ophthalmologists were also frequently consulted (on average 1.4 contacts per TTR-aHO-patient in year 1, 0.9 in year 2). Contacts with neurosurgeons, neurologists, radiologists, and gynecologists were also common in both follow-up years (see Supplemental Material Table S1). General practitioners were particularly involved in the outpatient follow-up of patients with TTR-aHO. Compared to patients with non-HO-obesity, patients with TTR-aHO had an average of + 4 more outpatient physician visits per quarter in the two quarters after the index event. In subsequent quarters, excess utilization gradually declined to + 2 contacts per quarter but remained significantly higher.

For patients with TTR-aHO, average number of prescribed pharmaceuticals remained relatively stable over time. However, in terms of excess utilization, patients with TTR-aHO had an average of over + 3 additional prescriptions per quarter compared to patients with non-HO-obesity.

**Table 2:** Average number of healthcare utilizations per patient with TTR-aHO and statistically matched non-HO obesity controls and related excess utilization based on a rolling 9-year cohort

Group	Index	Post observational period									
	QO	Q1	Q2	Q3	Q4	Q5	Q6	Q7	Q8	Y1	Y2
**Average number of hospitalizations**											
aHO	2.4	1.6	1.4	1.1	1.0	0.8	0.9	0.6	1.1	5.1	3.3
Non-HO-obesity	0.2	0.2	0.2	0.1	0.1	0.2	0.1	0.1	0.1	0.5	0.5
Excess	+ 2.2	+ 1.5	+ 1.2	+ 1.0	+ 0.9	+ 0.6	+ 0.8	+ 0.5	+ 0.9	+ 4.6	+ 2.8
**Average number of outpatient physician contacts**											
aHO	7.5	7.8	7.7	6.9	6.6	6.4	5.7	5.8	6.1	28.9	24.1
Non-HO-obesity	4.3	3.9	3.7	3.8	3.8	3.6	3.8	3.7	3.6	15.2	14.8
Excess	+ 3.2	+ 3.9	+ 3.9	+ 3.1	+ 2.9	+ 2.8	+ 2.0	+ 2.1	+ 2.5	+ 13.7	+ 9.3
**Average number of prescribed pharmaceuticals**											
aHO	4.0	5.8	5.5	5.7	5.5	5.5	5.3	5.4	5.8	22.5	22.1
Non-HO-obesity	2.4	2.4	2.4	2.3	2.3	2.3	2.3	2.2	2.2	9.3	9.0
Excess	+ 1.6	+ 3.4	+ 3.1	+ 3.5	+ 3.2	+ 3.3	+ 3.0	+ 3.2	+ 3.6	+ 13.1	+ 13.1

Q: Quarter, Y: Year

### TTR-aHO related costs

Mean overall costs during the index quarter of tumor surgery/radiotherapy (€25,717; SD: 33,995; Table 3) were higher in patients with TTR-aHO compared to patients with non-HO-obesity (€886; SD: 1,853), corresponding to total excess cost of +€24,800. Total excess costs remained high in the first year (+€19,900) and decreased in the second year (+€10,700) following the index quarter.

**Fig. 2:**
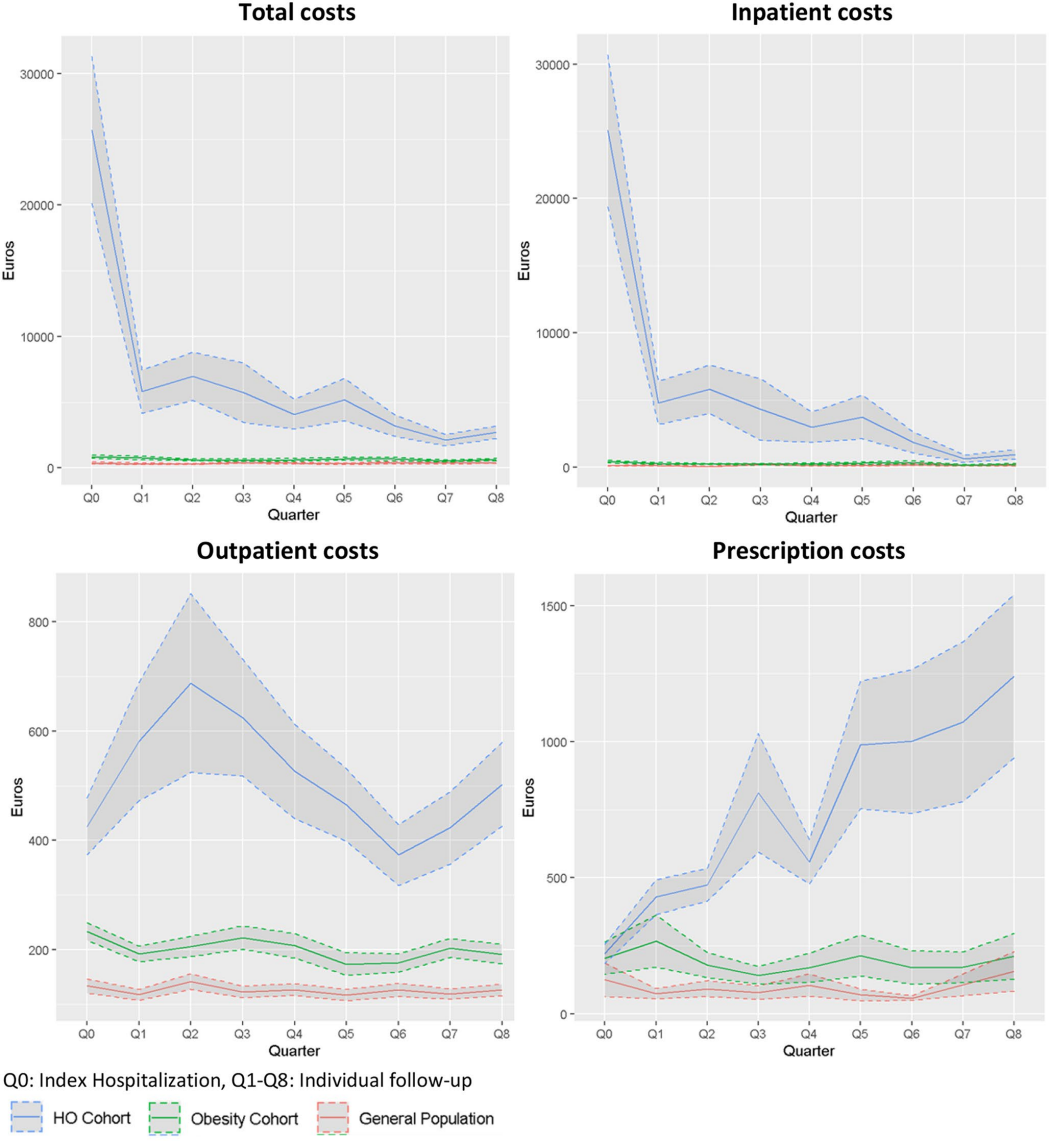
Treatment costs in patients with TTR-aHO compared to matched non-HO-obesity controls within two years after index hospitalization

In the TTR-aHO population, overall costs were largely driven by inpatient costs, i.e. follow-up hospitalizations after the tumor-related index hospitalization (Fig. 2). Mean excess inpatient costs per quarter decreased from the first year (+€4,100) to the second year (+€1,500) but remained higher than patients with non-HO-obesity.

Mean excess outpatient costs decreased slightly from the first year (+€1,592) to the second year (+€1,020) but remained elevated compared to patients with non-HO-obesity. On average, excess outpatient costs were +€398 per quarter in the first year and +€255 per quarter in the second year.

Average drug costs for patients with TTR-aHO were €2,276 in the first year after index hospitalization and almost 90% higher at €4,303 in the second year after index hospitalization (Table 3), corresponding to mean excess drug costs of +€1,518 in the first year and +€3,535 in the second year. On average, the quarterly drug expenditure of patients with TTR-aHO increased by 30% after index hospitalization within the two-year follow-up period. In contrast, there was no comparable cost trajectory for patients with non-HO-obesity (Fig. 2).

**Table 3:** Costs and excess costs [€] of patients with TTR-aHO compared to matched non-HO-obesity controls within two years after index hospitalization

Group	Index	Post observational period									
	QO	Q1	Q2	Q3	Q4	Q5	Q6	Q7	Q8	Y1	Y2
**Mean inpatient costs**											
aHO	25,071	4,793	5,798	4,302	2,997	3,744	1,844	623	962	17,890	7,173
Non-HO-obesity	420	294	233	230	248	306	341	150	233	1,004	1,030
**Mean outpatient physician costs**											
aHO	426	581	688	625	526	465	373	423	503	2,420	1,764
Non-HO-obesity	233	192	206	222	207	173	176	203	191	827	744
**Mean prescription costs**											
aHO	221	430	475	813	559	988	1,001	1,074	1,241	2,276	4,303
Non-HO-obesity	205	267	180	141	170	215	170	172	212	758	768
**Excess costs between patients with TTR-aHO and with Non-HO-obesity**											
Inpatient	24,566	4,534	5,237	4,115	2,739	3,495	1,469	493	777	16,624	6,154
Outpatient	193	389	482	402	319	292	198	220	311	1,592	1,020
Rx	16	163	294	672	389	773	831	902	1,029	1,518	3,535
Overall	24,832	5,194	6,078	5,200	3,463	4,563	2,498	1,615	2,099	19,935	10,745

Q: Quarter, Y: Year

[Note: Detailed cost information for patients with TTR-aHO and with non-HO-obesity, mean/median/SD, are provided in the Supplemental Material Tables S2-S4]

### TTR-aHO related prescription costs

A closer look at the costs of drug prescriptions shows that the costs per prescription for patients with TTR-aHO increased sharply over time, meaning that the observed cost trajectory was less about an increase in the number of prescriptions and more about an increase in cost-intensive prescriptions (Table 4).

**Table 4:** Average cost [€] per prescription [multiple prescriptions might apply] in aHO and matched patients with non-HO-obesity based on a rolling 9-year cohort

Group	Index	Post observational period									
	QO	Q1	Q2	Q3	Q4	Q5	Q6	Q7	Q8	Y1	Y2
aHO	58.0	65.2	92.3	160.6	101.5	223.2	199.1	368.3	288.0	107.4	246.6
Non-HO-obesity	45.8	70.9	45.6	40.1	41.1	69.0	43.3	38.9	43.4	53.2	50.0
Difference per prescription	+ 12.3	-5.7	+ 46.7	+ 120.5	+ 60.4	+ 154.2	+ 155.8	+ 329.4	+ 244.6	+ 54.1	+ 196.5

The observed increase in the cost of pharmaceuticals was due to rising prescription shares and the associated costs of hormone substitutes (Table 5). All patients with TTR-aHO were administered desmopressin within two years of their initial hospital stay, as per our inclusion criteria. Among all patients with TTR-aHO, 97% were given hydrocortisone, 27% received testosterone, and the same percentage received somatropin. The costs for somatropin were notably high.

**Table 5:** Utilization and costs [€] of hormonal replacement therapy in patients with TTR-aHO within two years after index hospitalization

Hormone replacement therapy	Index	Quarter after index hospitalization								
		Q1	Q2	Q3	Q4	Q5	Q6	Q7	Q8	SUM2Y
Hydrocortisone	64	84	77	88	76	83	137	111	157	605
Desmopressin	140	278	278	339	352	311	328	329	297	1.771
Levothyroxine	19	20	21	21	20	18	20	17	19	123
Somatropin	NA	NA	NA	NA	1.232	1.261	3.201	NA	3.368	6.811
Testosterone	NA	55	18	136	128	146	155	176	115	687
Dydrogesterone and estrogen	NA	NA	NA	34	NA	34	NA	NA	NA	212
Progesterone	NA	NA	NA	NA	50	NA	NA	29	NA	196
Estradiol	NA	NA	NA	NA	NA	NA	29	NA	58	128
Estriol	NA	NA	NA	NA	NA	NA	NA	NA	15	21

NA: Not applicable as no cases were observed. SUM2Y: Total share / mean costs within two years after index hospitalization

## Discussion

The resource utilization and costs of hypothalamic obesity are poorly understood because incidence and prevalence are related to several very rare underlying diseases. Our analyses indicate that TTR-aHO is associated with markedly higher HCRU than non-HO-obesity: TTR-aHO is associated with increased hospitalizations, increased outpatient physician visits, and increased, cost-intensive medication use. In terms of excess costs, inpatient costs comprised the largest share of overall excess costs in both years, primarily driven by follow-up hospitalizations, which may include costly neurosurgical interventions. However, prescription costs appear to represent a significant economic burden that grows over time. Average prescription costs increased substantially over the follow-up period, more than doubling the contribution of prescriptions to overall excess costs in the second year. This increase was due to cost-intensive hormone replacement therapies. As hormone replacement therapies are continuous, it is likely that prescription costs are an important driver of the long-term excess cost of TTR-aHO. In comparison to non-HO obesity, TTR-aHO represents a highly resource- and cost-intensive disease at the outset and over the long-term.

It is important to note that the analysis does not attempt to differentiate to what extent hormone replacement therapy is due to the tumor itself or to the development of hypothalamic obesity. In a clinical setting, patients with sellar/parasellar tumors are under close medical care and their symptoms and complications are treated wholistically. In practice, hormone replacement treatment is based on each patient’s individual profile, and physicians do not distinguish between tumor-related and obesity-related need for hormone replacement therapy.

Furthermore, for the analysis of excess costs, we also note that there was no use of relatively expensive GLP1R agonist therapies in the cohort; this is because therapies were not yet reimbursed by the statutory health system. However, in theory, a patient could have self-paid for a GLP1R prescription, but this would be unobservable in the data.

The identification approach in this study relies on three steps: incident inpatient surgery or radiotherapy (index event) following incident diagnosis of a tumor potentially leading to TTR-aHO; incident obesity diagnosis in the 12 months following the index event; and incident AVP-D and a desmopressin prescription within the 12 months following the index event. This approach may exclude patients with non-typical TTR-aHO trajectories, such as those who did not develop AVP-D or did not receive an obesity diagnosis within the 12 months following index event. However, although such a non-typical trajectory is possible, we believe the likelihood is low, as hypothalamic obesity is characterized by rapid weight gain and endocrine damage, which can be identified via AVP-D. Moreover, other research indicates that even patients with low basal weight can develop morbid obesity in a short period of time [22]. Therefore, we believe that the identification approach is conservative but reasonable. In addition, although the approach does not require the diagnosis of obesity to persist long-term, additional analysis indicates that the diagnosis remains for identified patients. However, given the claims data setting, is possible that obesity diagnosis is simply carried forward rather than thoughtfully re-coded at each visit.

In general, the goal of this identification approach was to provide a conservative estimate with a low risk of false positives [14]; therefore, although 37 patients is a relatively small number of patients, we believe that it represents an important lower bound of the TTR-aHO population. However, we note that the resulting estimates of economic burden should be interpreted with caution commensurate to the small sample size. Future research should critically test this identification approach in other databases and settings; this will be important in generating consensus around a standard definition for TTR-aHO. A standard definition will mitigate uncertainty in future epidemiological estimates and other analyses.

This study analyzes a cohort of patients who developed incident obesity following incident inpatient tumor diagnosis and inpatient brain surgery/radiotherapy against a cohort of patients with general obesity. Another possible comparison group could be patients who received an incident inpatient tumor diagnosis and incident brain surgery/radiotherapy, but did *not* develop incident obesity. Although this comparison would theoretically provide insight on the excess costs of obesity itself, this approach is associated with great uncertainty in a claims data setting, as it is difficult to build a robust comparison cohort based on the lack of a diagnosis code. Relying solely on claims data, we cannot exclude the possibility that obesity is not documented as a diagnosis or documented with a significant delay. Therefore, future research in other data settings should compare excess costs between tumor patients who do and do not develop TTR-aHO.

Comparative data from other analyses on the care and costs of patients with TTR-aHO are still lacking. Economic analyses of patients with one of the most common causes of TTR-aHO, craniopharyngioma, can therefore provide a comparative orientation. But even these studies are rare. Current data from the US addresses the initial treatment and surgery for craniopharyngioma yet lacks information on the subsequent follow-up expenses. Long-term follow-up has not been extensively reported for adult or pediatric populations with regard to HCRU payments and readmissions. Considering the frequent endocrine sequelae and complications following this procedure that may affect quality of life, reports of health care metrics are critical to inform procedural cost analysis, bundled payment considerations, and patient population health care burden. Dietz et al. reported emergency room readmissions after craniopharyngioma resection within 30 days of discharge in between 8% and 58% of cases, depending on the extent of endocrine and nonendocrine complications [23]. We observed a total of 18% of all patients with TTR-aHO with an TTR-aHO-attributable emergency room readmission within 90 days. Furthermore, at 24 months postoperatively, Dietz et al. observed higher rates of hospital readmissions and a higher number of medication refills for patients with endocrine and nonendocrine complications compared to those without complications [23]. These results are in line with our findings, which also imply that neuroendocrine complications, such as the development of hypothalamic obesity, are associated with higher resource use and higher costs. Dietz and colleagues use an exclusively adult cohort, and identify patients based on diagnosis of craniopharyngioma and skull base surgery procedure codes. This contrasts with our identification approach, which attempts to identify hypothalamic obesity in a cohort of all ages. Dietz and colleagues provide cost data for the US; however, comparing these findings is limited by variations in cost structures in the US and Germany.

Recent analyses of the costs of non-HO obesity in Germany estimate a substantial excess burden on social welfare systems and a significant amount of pain and suffering for affected individuals [24]. As we find that TTR-aHO is associated with significantly higher excess resource use and costs than non-HO-related obesity, it is likely that TTR-aHO represents an outsized cost burden on healthcare and social welfare systems. Due to the rapid course of TTR-aHO-related weight gain, patients likely experience a great deal of mental and physical suffering. The consistently high number of outpatient contacts and increasing share of hormone replacement therapies point to an ongoing, onerous, and expensive treatment journey long after the initial tumor-related hospitalization.

One of this study’s primary strengths is the large data set, including many patients with TTR-aHO, providing a comprehensive database for the validation of TTR-aHO through incident obesity, central AVP-D diagnosis, and desmopressin prescription. The dataset is generally representative with respect to age and gender. However, insured persons in the dataset are, on average, slightly younger than the overall German population and males are slightly overrepresented in the data. In terms of regionality, western and southwestern Germany are slightly overrepresented compared to eastern Germany. Assessment of the representativeness of the aHO population or the underlying tumor population is not possible due to lack of comparative data. Besides, the study design and methods face limitations. First, we are limited by the lack of information on the history of TTR-aHO-applied definition and validation criteria to define the incident study population prior to the observational period. If longer treatment intervals between hospitalizations with TTR-aHO occur, a 1-year wash-in period could be too short, which could lead to misclassified TTR-aHO incident patients. However, in the absence of a standardized definition of TTR-aHO, the chosen approach represents the best possible approximation of a TTR-aHO cohort for the evaluation of care and cohort characteristics. Future research should critically test this definition of TTR-aHO in other databases and settings. Second, as no clinical parameters such as BMI, laboratory test results, or information on health behavior are available in claims data, the analyses of weight gains are limited by the available diagnosis codes. Poor sensitivity of ICD-10 diagnosis data for administrative diagnosis of overweight/obesity has been reported in the literature [25]. However, it can be assumed that documentation might be more accurate because of the high patient relevance of rapid weight gain in TTR-aHO. Third, the potential for upcoding must be considered for some validation criteria [26]. This effect should, however, be small due to our strict case definition (i.e., in the primary analysis, all validation criteria need to be documented within 4 quarters after index hospitalization). Fourth, self-selection of patients is possible. For example, patients with severe weight gain might be more likely to seek treatment (and be documented in claims data) than patients with fewer health problems after TTR-aHO. However, this effect is probably small for the validation criteria applied in this study, as symptoms are typically severe and diagnosed by the physician, which makes the classification less likely to be influenced by self-selection. Finally, we apply a payer perspective on TTR-aHO resource utilization and costs and do not estimate the economic burden on patients and caregivers. One more limitation emerges from our exclusion of some cost components. Our assessment only includes direct disease-related to TTR-aHO or associated excess costs. We do not factor in indirect costs like lost earnings or informal caregiver burdens. A survey of eighty-two caregivers of craniopharyngioma survivors illustrates the unmet needs of craniopharyngioma survivors and their caregivers, associated with the survivor’s symptomatology impacting HRQOL [27].

Novel treatments in development for acquired HO such as setmelanotide are likely to further increase pharmacological costs and overall costs of care. This will need to be justified by reduction in disease burden to patients and caregivers, improvement in long-term outcomes and improvements in patient QoL.

## Conclusion

This is the first real-world database analysis of the economics of TTR-aHO. Analysis of HCRU and costs of rare diseases like TTR-aHO is highly important—not only to understand the disease, but also to inform regulatory and health-economic decision making. Due to lack of definitional standards for clinical documentation, identification of patients with aHO in previous retrospective studies has been associated with an uncertainty which also extends to the associated economic evaluations. In this study, we use a newly refined definition from Witte et al. [14] which enables a reliable estimation of the economic impact of the disease. Our findings indicate a significant strain on the healthcare system for patients with TTR-aHO. Patients with TTR-aHO have significantly elevated healthcare use in all sectors compared to patients with non-HO-obesity. The economic burden of TTR-aHO is characterized by high excess inpatient costs due to follow-up hospitalizations, consistently elevated excess outpatient costs, and a dramatic increase in excess prescription costs in the second year after the index event. Prescription costs are driven by the need for ongoing hormone replacement therapies, which lead to a continuous rise in medication costs and additional drug safety management for patients with TTR-aHO within two years of tumor surgery or radiotherapy. Future research should focus on the development of new treatments and cost reduction strategies for TTR-aHO.

## Electronic supplementary material

Below is the link to the electronic supplementary material.

Supplementary Material 1

## Data Availability

Project-specific access to an anonymized, selected study data set for the analyses was provided by the GWQ ServicePlus AG. The data analyzed in this study are not publicly available due to data protection regulations and national legislation. All data-related processes of Vandage are under the data protection supervision of an external data protection officer.

## Declarations

### Medical writing, editorial, and other assistance

J.W., K.B., B.S., H.L.M, J.B., and N.T. prepared the manuscript. All other co-authors reviewed and approved the manuscript. Medical writing/editing support was provided by Excerpta Medica, funded by Rhythm Pharmaceuticals.
