# Supplementary material for: Economics of hypothalamic obesity in patients with craniopharyngioma and other rare sellar/suprasellar tumors
Source: Eur J Health Econ. 2025 May 9;26(9):1557–67. doi: 10.1007/s10198-025-01786-3 (PMC12618323; doi:10.1007/s10198-025-01786-3)
Supplement: Supplementary file 1 — Supplementary Material 1 [file 10198_2025_1786_MOESM1_ESM.docx]

Supplementary Appendix

Contents

[**Table S1. Outpatient contacts (mean per patient) with specialist groups within the two years following TTR-HO-associated index hospitalization** 2](#_Toc166662994)

[**Table S2. Inpatient costs of patients with TTR-HO compared to matched non-HO-obesity controls within two years after index hospitalization (detail)** 3](#_Toc166662995)

[**Table S3. Outpatient costs of patients with TTR-HO compared to matched non-HO-obesity controls within two years after index hospitalization (detail)** 4](#_Toc166662996)

[**Table S4. Prescription costs of patients with TTR-HO compared to matched non-HO-obesity controls within two years after index hospitalization (detail)** 5](#_Toc166662997)

# **Table S1. Outpatient contacts (mean per patient) with specialist groups within the two years following TTR-HO-associated index hospitalization**

| **Specialist group** | **Index** | **Post observational period** | | | | | | | | | |
| --- | --- | --- | --- | --- | --- | --- | --- | --- | --- | --- | --- |
|  |  | **Q1** | **Q2** | **Q3** | **Q4** | **Q5** | **Q6** | **Q7** | **Q8** | **Y1** | **Y2** |
| General Practitioner | 1.65 | 1.59 | 1.84 | 1.62 | 1.54 | 1.54 | 1.22 | 1.46 | 1.46 | 6.59 | 5.68 |
| Endocrinology/Diabetology | 0.27 | 0.38 | 0.38 | 0.32 | 0.35 | 0.27 | 0.27 | 0.22 | 0.32 | 1.43 | 1.08 |
| Opthamology | 0.49 | 0.49 | 0.19 | 0.35 | 0.35 | 0.27 | 0.22 | 0.16 | 0.24 | 1.38 | 0.89 |
| Gynacology | 0.22 | 0.16 | 0.27 | 0.22 | 0.24 | 0.38 | 0.24 | 0.27 | 0.30 | 0.89 | 1.19 |
| Neurosurgery | 0.19 | 0.24 | 0.11 | 0.14 | 0.08 | 0.08 | 0.03 | 0.03 | 0.14 | 0.57 | 0.28 |
| Neurology | 0.19 | 0.08 | 0.16 | 0.19 | 0.14 | 0.08 | 0.16 | 0.08 | 0.14 | 0.57 | 0.46 |
| Radiology | 0.24 | 0.22 | 0.05 | 0.22 | 0.05 | 0.19 | 0.05 | 0.05 | 0.16 | 0.54 | 0.45 |
| Oncology/Haematology | 0.00 | 0.00 | 0.03 | 0.00 | 0.00 | 0.00 | 0.00 | 0.00 | 0.00 | 0.03 | 0.00 |
| Other | 1.13 | 1.16 | 1.52 | 1.18 | 1.25 | 1.43 | 1.19 | 1.52 | 1.48 | 5.11 | 5.62 |
| Q: Quarter; Y: Year  Other: Includes less-utilized specialties such as dermatology, otorhinolaryngology, gastroenterology, and cardiology. | | | | | | | | | | | |

# **Table S2. Inpatient costs of patients with TTR-HO compared to matched non-HO-obesity controls within two years after index hospitalization**

|  | **Index** | **Post observational period** | | | | | | | | | |
| --- | --- | --- | --- | --- | --- | --- | --- | --- | --- | --- | --- |
| **Group** | **QO** | **Q1** | **Q2** | **Q3** | **Q4** | **Q5** | **Q6** | **Q7** | **Q8** | **Y1** | **Y2** |
| **Inpatient costs** | | | | | | | | | | | |
| **HO** | | | | | | | | | | | |
| Mean costs | 25,071 | 4,793 | 5,798 | 4,302 | 2,997 | 3,744 | 1,844 | 623 | 962 | 17,890 | 7,173 |
| Median costs | 13,466 | 263 | 400 | 189 | 49 | 0 | 98 | 0 | 192 | 3,707 | 1,295 |
| Std. dev. | 34,129 | 9,865 | 10,990 | 13,887 | 6,935 | 9,967 | 4,808 | 1,739 | 2,230 | 29,095 | 14,325 |
| **Non-HO-obesity** | | | | | | | | | | | |
| Mean costs | 420 | 294 | 233 | 230 | 248 | 306 | 341 | 150 | 233 | 1,004 | 1,030 |
| Median costs | 0 | 0 | 0 | 0 | 0 | 0 | 0 | 0 | 0 | 0 | 0 |
| Std. dev. | 2,123 | 1,500 | 987 | 1,196 | 1,646 | 1,927 | 2,330 | 780 | 1,135 | 2,904 | 4,299 |
| Q: Quarter, Y: Year | | | | | | | | | | | |

# **Table S3. Outpatient costs of patients with TTR-HO compared to matched non-HO-obesity controls within two years after index hospitalization**

|  | **Index** | **Post observational period** | | | | | | | | | |
| --- | --- | --- | --- | --- | --- | --- | --- | --- | --- | --- | --- |
| **Group** | **QO** | **Q1** | **Q2** | **Q3** | **Q4** | **Q5** | **Q6** | **Q7** | **Q8** | **Y1** | **Y2** |
| **Outpatient costs** | | | | | | | | | | | |
| **HO** | | | | | | | | | | | |
| Mean costs | 426 | 581 | 688 | 625 | 526 | 465 | 373 | 423 | 503 | 2,420 | 1,764 |
| Median costs | 349 | 472 | 448 | 390 | 396 | 346 | 253 | 267 | 421 | 1,667 | 1,401 |
| Std. dev. | 316 | 658 | 996 | 650 | 527 | 404 | 338 | 404 | 467 | 2,022 | 1,329 |
| **Non-HO-obesity** | | | | | | | | | | | |
| Mean costs | 233 | 192 | 206 | 222 | 207 | 173 | 176 | 203 | 191 | 827 | 744 |
| Median costs | 136 | 109 | 102 | 96 | 98 | 93 | 93 | 115 | 92 | 488 | 483 |
| Std. dev. | 308 | 279 | 360 | 418 | 427 | 392 | 318 | 336 | 336 | 1,192 | 1,191 |
| Q: Quarter, Y: Year | | | | | | | | | | | |

# **Table S4. Prescription costs of patients with TTR-HO compared to matched non-HO-obesity controls within two years after index hospitalization**

|  | **Index** | **Post observational period** | | | | | | | | | |
| --- | --- | --- | --- | --- | --- | --- | --- | --- | --- | --- | --- |
| **Group** | **QO** | **Q1** | **Q2** | **Q3** | **Q4** | **Q5** | **Q6** | **Q7** | **Q8** | **Y1** | **Y2** |
| **Prescription costs** | | | | | | | | | | | |
| **HO** | | | | | | | | | | | |
| Mean costs | 221 | 430 | 475 | 813 | 559 | 988 | 1,001 | 1,074 | 1,241 | 2,276 | 4,303 |
| Median costs | 149 | 335 | 376 | 508 | 419 | 492 | 528 | 539 | 562 | 2,094 | 2,506 |
| Std. dev. | 212 | 389 | 366 | 1,328 | 497 | 1,430 | 1,611 | 1,783 | 1,827 | 1,538 | 5,151 |
| **Non-HO-obesity** | | | | | | | | | | | |
| Mean costs | 205 | 267 | 180 | 141 | 170 | 215 | 170 | 172 | 212 | 758 | 768 |
| Median costs | 31 | 23 | 27 | 25 | 24 | 23 | 23 | 17 | 23 | 130 | 111 |
| Std. dev. | 1,141 | 1,838 | 883 | 634 | 1,037 | 1,463 | 1,182 | 1,085 | 1,619 | 3,312 | 5,056 |
| Q: Quarter, Y: Year | | | | | | | | | | | |
